# Genetic and epigenetic biomarkers in human biomonitoring: why needed and how can Oxford Nanopore sequencing contribute?

**DOI:** 10.3389/fpubh.2025.1610248

**Published:** 2025-07-01

**Authors:** Mathieu Gand, Adelheid Soubry, Birgit Mertens, Nancy H. C. Roosens, Sigrid C. J. De Keersmaecker

**Affiliations:** ^1^Transversal Activities in Applied Genomics, Sciensano, Brussels, Belgium; ^2^Epigenetic Epidemiology Lab, Department of Human Genetics, Faculty of Medicine, KU Leuven, Leuven, Belgium; ^3^Risk and Health Impact Assessment, Sciensano, Brussels, Belgium

**Keywords:** susceptibility biomarker, effect biomarker, SNP, DNA-methylation, human biomonitoring, Oxford Nanopore Technologies, large-population study

## Abstract

Chemical risk assessment can benefit from integrating informative biomarkers in human biomonitoring (HBM). Beyond exposure biomarkers, effect biomarkers inform on biological reactions in the body, potentially leading to adverse effects, while susceptibility biomarkers address inter-individual variability in exposure. DNA methylation of key genes shows promise as an effect biomarker but this epigenetic mark remains underexplored in the context of chemicals. Similarly, although some genetic polymorphisms are linked to increased chemical susceptibility, genetic biomarkers are rarely included in HBM. This mini-review highlights recent literature supporting the inclusion of genetic and epigenetic biomarkers in HBM. Subsequently, we elaborate on how Oxford Nanopore Technologies as sequencing method can efficiently measure these biomarkers simultaneously, even in non-invasive samples like saliva. Widely used in other fields, this experimental set-up could facilitate the design of large-population studies paving the way for a next generation risk assessment (NGRA) of chemicals.

## 1 Introduction

Some chemicals present in the environment have a high toxicological profile (e.g., bisphenols, phthalates, perfluoroalkyl substances, heavy metals and air pollution) and may have an impact on human health, with adverse effects at the biochemical, physiological, and/or behavioral level, potentially leading to diseases (e.g., cancer, neurodegeneration or reproduction/growth disorder) (1, 2). It is important to clearly evaluate the risks linked to these chemicals, so that prevention and mitigation actions can be taken. Risk assessment is generally based on results of *in vitro* and *in vivo* (animal models) toxicity tests, epidemiological data, and by measuring the presence of chemicals in the environment (1, 3, 4). However, this does not give the full picture, as the internal exposure and the subsequent adverse effects in the human body are as such not accurately evaluated (3, 4). To address this, human biomonitoring (HBM) measures chemicals and their related biomarkers directly in the organism, and can therefore be an important source of additional and more accurate information from a public health and regulatory perspective (2, 4). Biomarkers for HBM have various types and applications. For instance, the exposure biomarkers, such as urinary levels of phthalate metabolites (5, 6), inform about the internal and biological effective dose of chemicals absorbed by the body (7). In contrast, effect biomarkers reflect early biological responses to chemical exposure, evidencing altered molecular/cellular structures and functions such as DNA alteration, enzyme induction/inhibition, elevated hormone levels or altered metabolic parameters, which may precede the onset of symptoms (7). As such, the exposure can be connected to human health alteration, potentially leading to disease. These effect biomarkers are thus informative for risk assessment, because they can be linked to toxicological effects, thus helping to construct Adverse Outcome Pathways (AOP), as conceptualized by the European Human Biomonitoring Initiative (HBM4EU) working groups (7) and extended in the Horizon Europe funded Partnership for the Assessment of Risks from Chemicals (PARC)^1^ (8). However, for a same level of external exposure, the level of internal exposure and effect biomarkers can vary in the population, due to differences in the biological reactions of the organism, influenced by genetic polymorphisms in the human genome (9), as it was already demonstrated for Bisphenol A (BPA) (10) and lead (Pb) (11). To account for this variability, susceptibility genetic biomarkers, such as specific Single Nucleotides Polymorphisms (SNPs) in genes involved in adsorption, distribution, metabolism and excretion of chemicals, can be measured to identify people who are potentially more susceptible to some adverse effects (11). Ideally, exposure, effect and susceptibility biomarkers should be combined in HBM for accurate and improved next generation risk assessment (NGRA) (7, 11).

A promising prototype effect biomarker is an epigenetic one, i.e., DNA-methylation (DNAm) of key genes associated with adverse outcomes due to chemical exposure (12). The hyper-methylation of DNA, especially in regions rich in CpG dinucleotides (CpG islands) and located in genes’ promoter regions, is known to decrease gene expression, while hypo-methylation has the opposite effect (13). Modifications, triggered by environmental exposure, can occur in DNAm levels of CpG islands and this can affect how the genes are expressed through epigenetic mechanisms (1, 14, 15). These epigenetic modifications can be maintained in the body for a life time, and, if occurring in germ cells, even be transmitted to offspring as transgenerational marks (16). A tremendous number of studies on epigenetics have demonstrated the key role of DNAm in cancer and aging, and this biomarker is now used in precision medicine for diagnostic, prognostic and disease prediction (17, 18). Therefore, DNAm has also the potential to be used as an effect biomarker in HBM studies for chemical risk assessment (1, 12, 19). In addition to DNAm, epigenetics encompasses also other layers including histone modification or non-coding RNAs (ncRNAs), which might be used as effect biomarkers in HBM (1, 20). However, here we focused on DNAm as it is currently the most feasible mark for HBM.

The most common method for DNAm profiling relies on short-read Next Generation Sequencing (NGS) with the Illumina technology, requiring DNA chemical pre-treatment (bisulfite) and enrichment using arrays (21). In the past few years, third-generation sequencing techniques, such as Oxford Nanopore Technologies (ONT), aroused interest as they can simultaneously perform raw sequencing of long DNA fragments and detect base modifications such as methylation, without pre-treatment (18). During ONT sequencing, the ionic current change is measured while the DNA strands pass through small protein channels (nanopores) in a flowcell (MinION or PromethION). These variations in the electric signal are converted into the four nucleotide bases, as well as their modifications (22), through machine learning algorithms (basecalling). Among these modifications, cytosine methylation is included, but without being limited to it. This technology would be ideal to measure both DNAm and SNP-based biomarkers in HBM studies.

To have a strong impact and produce robust evidence, HBM studies should ideally measure informative biomarkers at multiple levels and time points, and encompass a high number of participants. However, the design of large-population studies raises a certain number of challenges in terms of cost and practical organization, which can limit the number of participants and biomarkers investigated. To face these challenges, the choice of biomarkers and analytical methods are critical (4). In this mini-review, we advocate for the inclusion of the measurement of epigenetic (DNAm) and genetic (SNP) marks in HBM studies, as effect and susceptibility biomarkers, for improved chemical risk assessment. DNAm biomarkers showed their potential in other fields, but are still understudied in the context of environmental exposure. The same observation holds true for susceptibility biomarkers which are not often included in HBM. Furthermore, we highlight the use of ONT for a simultaneous detection of SNPs and DNAm. Finally, we discuss how the advantages brought by this technology for combined straightforward genotyping and base modification detection, have the potential to address the aforementioned challenges inherent to large-population studies. To support our proposal, we included Table 1 with some examples of HBM studies in the context of chemical exposure, investigating DNAm alterations (with or without SNPs typing) including a brief description of the methods employed for biomarker detection.

**Table 1:** Examples of epigenetic and genetic biomarkers, and the methods to detect them, in association with chemical exposure in HBM studies.

Study info				Epigenetic biomarkers							Genetic biomarkers		Ref
Chemical	Nbr subj	Subj type	Sample	DNAm alteration	Marker type	Methylation detection	Bisul conv	Cell type	Pathw analy	Gene expr	SNPs	Genotyp method	
Arsenic^uri^	202	Women (15 - 64)	P blood	Hyperm *p16* and *MLH1*	Canc	Pyroseq.	Yes	ND	No	No	*AS3MT* haplotype 1	Sequenom MassArray	(28)
Arsenic^ext^	16	Adults (30 - 70)	P blood	Hypom *NAPRT1*, *NT5C3B*, and *NEDD4L*; Hyperm *RAB11B* and *SLC22A3*	Spec	Infinium 850 k + seq.	Yes	ND	Yes	Yes	No	-	(84)
Arsenic^uri, ext^	102	Children (5 - 16)	P blood	Hyperm *MLH1* and *MSH2*; Hypom *TFAM* and *PGC1*α	Spec, canc	MSP	Yes	ND	No	No	No	-	(30)
Benzene^ext^	8	Adult workers (34 - 55)	P blood	Hyperm *PRKG1*, *PARD3*, and *EPHA8*; Hypom *STAT3* and *IFNGR1*	Spec, canc	Infinium 450 k + seq.	Yes	Meas	Yes	Yes	No	-	(85)
Benzene^ext^	98	Adult workers (mean 30)	P blood	Altered DNAm in AMPK signaling pathway genes	Spec	Infinium 450 k + seq	Yes	Estim	Yes	No	No	-	(86)
BPA^uri^	6 & 146	Mother - infants (birth)	Placenta tissue	Hyperm *HLA-DRB6*	Spec	Infinium 450 k + seq & pyroseq	Yes	ND	Yes	No	No	-	(87)
BPA^uri^	309, 156 & 150	Mother - infants (birth, 9 & 14)	C & P blood	Hyperm *GRIK1*, *CXCL14*, *OXTR* and *AK7*; altered DNAm *RNF39*	Spec	Infinium 450 k + seq	Yes	Estim	Yes	No	No	-	(88)
Lead (Pb)^uri^	27	Adult men (20 - 40)	Sperm	Altered DNAm in neurological system, cytoskeleton and Ca pathway genes	Spec	MeDIP + seq	No	Meas	Yes	No	No	-	(36)
Lead (Pb)^blo^	30 & 305	Adult workers (med 33 & 38.5)	P blood	Hypom *RRAGC* and *USP1*	Spec	Infinium 450 k + seq & pyroseq	Yes	Estim	Yes	No	No	-	(89)
Flame-retardant^uri^	67	Adult men (18 - 35)	Sperm	Hyperm *NDN*, *SNRPN* and *GRB10* Altered DNAm of *MEG3* and *H19*	Spec, canc	Pyroseq.	Yes	Meas	No	No	No		(31)
Pesticides^ext^	1,170	Male workers (55 - 72)	P blood	Altered DNAm in 162 CpGs across 8 active ingredients	Spec	Infinium 850 k + seq.	Yes	Estim	Yes	Yes	No	-	(33)
Pesticides^ext^	218, 222, 572 & 2,333	Adults (18 – 75)	P blood	Altered DNAm in 50 genes involved in, e.g., cell signaling, transcription, translation, polyubiquitination, and ion/nucleotide transporters	Spec	Infinium 450 k & 850 k + seq	Yes	Estim	No	No	PD’s SNPs + SNPs influencing DNAm	Illumina NeuroChip array	(42)
PFAS^blo^	141	Mother - infants (birth)	C blood	Hypom of intergenic region chr 22; Hyperm of *ATG2A*, *GTPBP3* and intergenic region chr 5	Spec	Infinium 850 k + seq.	Yes	Estim	Yes	Yes	No	-	(90)
PFAS^blo^	63	Children (7 - 11)	P blood	Hypermethylation of *RASAL2* and *ITPR1* Hypomethylation of *MCF2L*,	Spec	Infinium 850 k + seq.	Yes	Estim	Yes	No	No	-	(91)
PFAS^blo^	182	Male workers (25 - 49)	P blood	Altered DNA methylation in *HAS2-AS1*, *NSMCE2*, *LINC00824*, *FAM49B*, *ASAP1* and intergenic regions in chromosome 8.	Spec	Infinium 450 k + seq & pyroseq	Yes	Estim	No	No	No	-	(61)
Phtalates^uri^	64	Mother - infants (birth)	C blood	Altered DNAm in *PA2G4*, *HMGCR* and *XRCC6*	Spec	Infinium 450 k + seq & pyroseq	Yes	Estim	Yes	No	No	-	(6)
Phtalates^uri^	74 & 78	Mother - infants (mean 2.88 months)	P blood & buccal cells	Altered DNAm in endocrine hormone activity, immune pathways, DNA damage and neurodevelopment genes	Spec	Infinium 450 k + seq	Yes	Estim	Yes	No	No	-	(5)

The table lists examples of DNA-methylation (DNAm) and Single Nucleotide Polymorphism (SNP) biomarkers identified in human biomonitoring (HBM) studies in context of exposure to chemicals. The exposure to chemicals was assessed either by measurement of internal exposure biomarkers in urine (^uri^) or blood (^blo^), or by external exposure (^ext^) estimation. The type of subjects (“subj type”) is followed by the age range (in years if not stipulated) of the study population between brackets. When the range was not communicated, the age mean or median (“med”) is given. In case of mother - infants pairs, chemical exposure was assessed for the mother during pregnancy and DNAm was measured for the infant at birth and/or in the first months/years of life. “Samples” to measure effect biomarkers were mostly blood from peripheral (“P”) or cord (“C”) origin. “DNAm alterations” were detailed for key genes that can potentially be used as effect biomarkers. If no key genes were highlighted by the study authors or if they were too many to be listed in the table, information about the potentially impacted biological functions is given instead. The DNAm biomarkers were classified as being broadly related to cancer/tumors (“canc”) or specific (“spec”) of the investigated exposure. DNAm was detected with PCR followed by pyrosequencing (“pyroseq”), with methylation specific PCR (“MSP”) followed by electrophoresis, with Methylated DNA immunoprecipitation (“MeDIP”) followed by Illumina sequencing (“seq”), with Infinium HumanMethylation450 (“Infinium 450 k”) or with Infinium MethylationEPIC (“Infinium 850 k”) arrays followed by Illumina sequencing (“seq”). Cellular variability (“cell type”), influencing DNAm measurement, was accounted for by experimental measurement (“meas”) of the sample cellular composition, by estimation (“estim”) using DNAm data and deconvolution methods, or was not determined (“ND”). Some studies conducted pathway analysis (“pathw”) using gene annotation and/or enrichment with for instance Gene ontology or Kyoto encyclopedia of genes, to link identified DNAm alteration with biological functions. Gene expression (“Gene expr”) analysis was sometimes performed to investigate if DNAm alterations could be linked to modification in transcription of the corresponding genes. In the studies (87) and (89), EWAS was performed on a small subset (first number in “Nbr subj”) of the study population using Infinium arrays and later replicated in an independent experiment with more subject (second number in “Nbr subj”) using pyrosequencing. In study (42), replication of the results was performed with publicly available DNAm data (the 3 last numbers in “Nbr subj”). In the same study, monogenic mutations associated with Parkinson disease (PD’s SNPs) were measured. In study (88), the 3 numbers in “Nbr subj” corresponds, respectively, to the infants followed at birth, 9 and 14 years old. In study (61) pyrosequencing was used for a better resolution of repetitive elements (not possible with Infinium arrays). In study (5), the 2 numbers in “Nbr subj” correspond to the number of participants for which peripheral blood and buccal cells have been collected for comparison in this study, respectively.

Nbr: number; subj: subject; Bisul conv: bisulfite conversion; periph blood: peripheral blood; Hyperm: hypermethylation; hypom: hypomethymation; cord blood: umbilical cord blood; chr: chromosome; Genotyp: genotyping.

## 2 DNA-methylation: an informative effect biomarker to combine with genetic background

Ideally, effect biomarkers should meet the following criteria: being predictive (biologically relevant), specific (discriminative) and sensitive (reliable measurement) (7, 23). Several DNAm alterations have been associated to exposure and proved to be valid candidates for effect biomarkers (1, 12).

DNAm levels of naturally hypermethylated transposable repetitive elements (e.g., LINE-1 and Alu (24–27)) or tumor suppressor genes (e.g., *CDKN2A (p16)* and *CDKN2B (p15)* (24, 28, 29)) and oncogenes (e.g., *MAGE-A1*, *H19*, *MLH1* and *MSH2* (26, 28, 30, 31)) were historically used to inform about global methylation modifications in the body, and to make the link between exposure and cancer development. This was for instance documented in a study reporting global methylation changes in blood associated with air pollution (32). However, these markers were too general and can be associated to various causes. Other studies succeeded to establish a more specific link between exposure and DNAm alterations, thus identifying informative biomarkers. This was for instance the case with exposure to phthalates associated with DNAm in 12 high-confidence CpGs (5), with active ingredients from pesticides associated with unique differentially methylated CpGs (33) and DNAm alteration of the *AHRR* gene in the context of tobacco-smoking (34, 35). These associations were sometimes replicated with independent analyses using a different study population, confirming the discriminatory power of the biomarker candidates (Table 1).

DNAm is linked to gene expression and can explain the early biological mechanisms triggered by chemicals. Using gene annotation, gene enrichment, pathway and gene expression analyses, it is possible to make the link between differential DNAm of genes involved in key cellular functions, and the adverse effects associated with exposure (Table 1). For instance, in studies about sperm quality in relation with lead and phthalates exposure (36, 37), DNAm alterations were identified in genes involved in cytoskeleton formation, which plays a critical role in sperm motility and fertilization. Another study identified DNAm patterns in genes related to endocrine activity in subjects exposed to phthalates, known to interfere with hormonal regulation (5). Establishing a link with biological effects is even more critical in epigenome-wide association studies (EWAS), where dozens to hundreds of differentially methylated regions are reported, and the most relevant ones must be identified. Although these biological associations need further validation at the analytical, toxicological, and physiological levels, they help to select informative effect biomarkers based on their biological relevance, and contribute to the AOP.

The DNAm alterations in response to chemicals can vary in the population because of inter-individual genetic variability (38). Indeed, particular SNPs were associated with slower metabolization of chemicals (39, 40), resulting in higher internal dose of metabolites in the body and prolonged adverse effects, as it was demonstrated for BPA (10) and lead (Pb) (11). Moreover, genetic polymorphisms can influence how the body reacts to exposure and how biomarkers are produced (41). Finally, some people have higher chances to develop non-communicable diseases because of their genetic background (42). Several studies and reviews listed genetic variations associated with a higher susceptibility to a.o. arsenic (43), lead (Pb) (11, 44), phthalates (45), mercury (46), benzene (47) and pesticides (48, 49). This genetic variability introduces potential bias and confounding factors in the measurement of exposure and effect biomarkers. Additionally, SNPs in metabolism-related genes are particularly of health concern as they can increase the duration of the adverse effects. While it appears relevant to include genotyping of susceptibility biomarkers in HBM for more accurate interpretation, we found only two DNAm studies including SNPs measurement in this context (Table 1). In one study (28), genotyping of *AS3MT* was performed in parallel with DNAm analysis, as its haplotype 1 is constituted of several polymorphisms associated with a slower arsenic metabolism. The genetic component of Parkinson disease was taken into account in another DNAm study (42), by excluding participants having higher chances to develop the disease because of specific polymorphisms, and investigating for the remaining ones the cross effect of genetic background and pesticide exposure on DNAm. As shown in this section, several associations are being established in the scientific literature between genetic polymorphisms (in coding and non-coding regions) and susceptibility to chemicals. Even more genetic data is available in the genome wide association study (GWAS) catalog and will continue to be added (50). Therefore, including genotyping of critical SNPs (selected from existing publicly available data) in HBM should be considered, to account for variation in people’s susceptibility and internal exposure dose, and combined with other informative biomarkers such as DNAm, to contribute to improved risk assessment. To achieve this, new methods are required for adequate and routine inclusion of genotyping in DNAm studies, as already suggested almost a decade ago in the context of smoking DNAm biomarkers influenced by SNPs (35).

## 3 The ideal technique for measuring both genetic and epigenetic markers: Oxford Nanopore sequencing holds promise

The genotyping methods for SNP analysis are well established and were commonly used in studies investigating for genetic predisposition to non-communicable diseases. If few polymorphisms are targeted, they can be detected using real-time polymerase chain reaction (qPCR) (41). If more targets are to be screened for, arrays are preferably used (42). Finally, for new marker discovery in GWAS, high-throughput sequencing methods are chosen for an untargeted approach (51).

The detection of DNAm requires other techniques, which in comparison with genotyping, are usually less straightforward and involve several steps. Indeed, the most common procedure for the detection of methylated CpGs consists of a chemical DNA treatment with bisulfite, to convert unmethylated cytosines into uraciles (later transformed into thymines following subsequent PCR) while the methylated cytosines remain unchanged. Subsequent detection techniques are then used to discriminate the converted cytosines from the unconverted ones. Among those techniques, methylation-specific PCR (MSP) followed by electrophoresis, or PCR followed by pyrosequencing, can be used if few CpGs are targeted (21, 52). Nowadays, hybridization capture with Illumina DNA methylation BeadChips followed by short-read high-throughput sequencing, enriching for more than 450,000 (Infinium HumanMethylation450) or 850,000 (Infinium MethylationEPIC) CpG sites (52) and covering 96% of the human CpG islands, is usually preferred to maximize the number of detected targets, especially in EWAS (Table 1). Nevertheless, the aforementioned sequencing techniques and the MSP have one major drawback coming from the bisulfite conversion. This chemical treatment can be harsh, introducing DNA damage, and does not lead to a 100% DNA conversion, leading to variability and hampering the accuracy of the method (53). As an alternative, selective enrichment of CpGs can be performed with Methylated DNA immunoprecipitation (MeDIP), which will capture the methylated DNA using specific antibodies (52, 53), allowing selective sequencing of methylated sites (Table 1). Although easy, specific and sensitive, this method can only identify the overall methylation status and is biased toward rich CpGs regions, requiring normalization.

**Figure 1:**
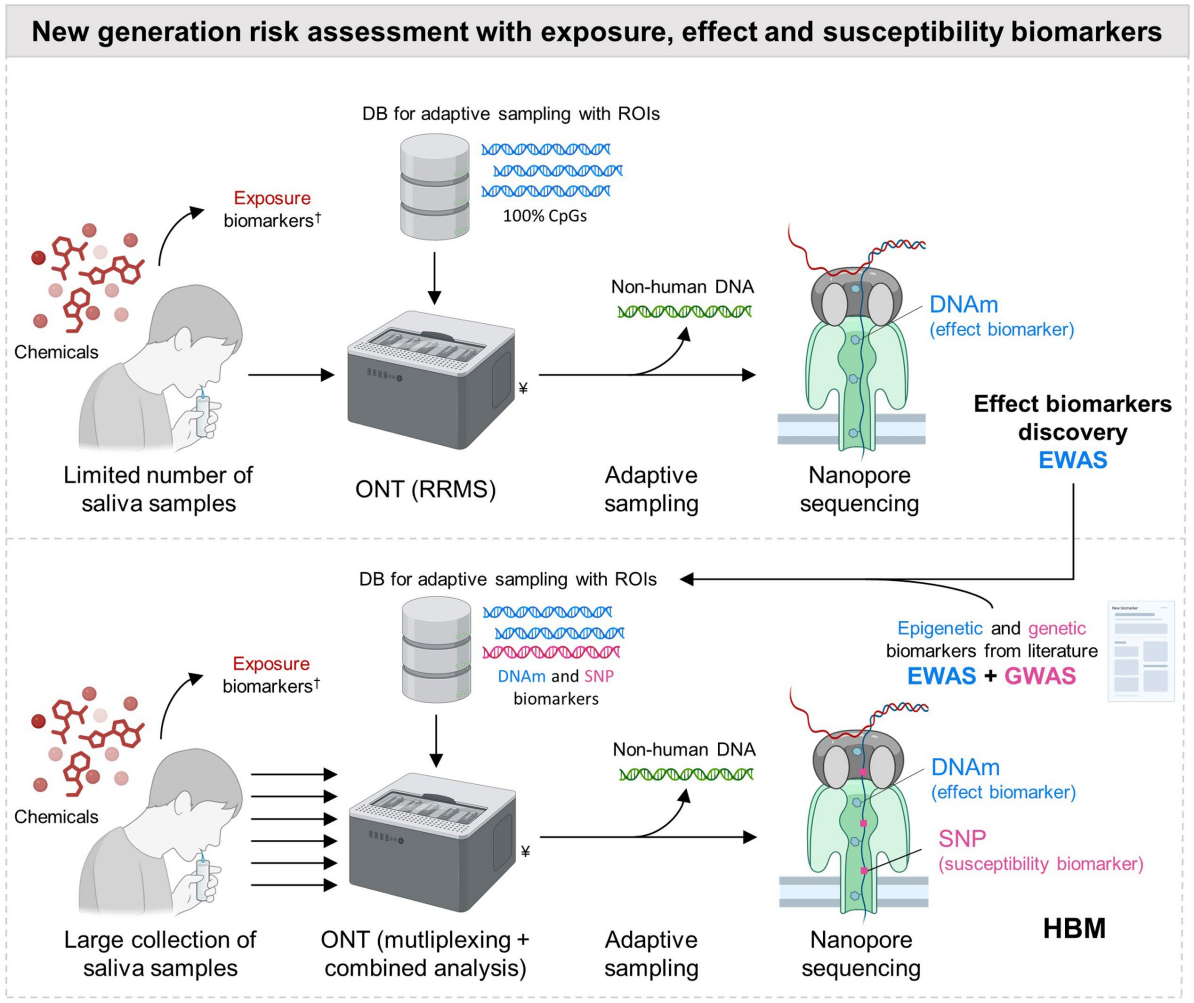
Next generation chemical risk assessment with Oxford Nanopore Technologies for analysis of genetic (SNP) and epigenetic (DNAm) biomarkers from non-invasive samples in HBM. Oxford Nanopore Technologies (ONT) is suggested for the simultaneous analysis of DNA-methylation (DNAm) and Single Nucleotide Polymorphisms (SNPs) from non-invasive samples (e.g., urine, semen, etc., and illustrated for saliva) for cost efficiency and facilitating organization and design of human biomonitoring (HBM) studies with large populations. “Adaptive sampling included,” in the Reduced-Representation Methylation Sequencing (RRMS) from ONT, consists of the real-time comparison of the 400 first bases sequenced in the nanopore, with Regions of Interest (ROI) stored in a database (DB). If these first bases match a ROI, the rest of the DNA strand is sequenced. If not, the strand is rejected from the pore allowing another DNA fragment to be tested. With the by default RRMS protocol from ONT including targeted ROI, this allows for the enrichment for 100% of the human CpGs, with depletion of non-human bacteria from the salivary microbiome, leading to accurate and high coverage detection of DNA-methylations. Nonetheless, this technology is scalable as the ROI stored in the DB can be customized by the user. As such, selective enrichment for SNPs can also be done if target polymorphisms are included as ROI in the DB. To maximize the use of the flowcell’s sequencing resources, a two-step strategy is suggested. The default RRMS protocol targeting 100% of the CpGs can be used in first for epigenetic biomarker discovery in EWAS setting, but with the limit of one sample per sequencing MinION flowcell, which can be costly but is needed to guarantee sufficient coverage for data analysis. The adaptive sampling DB can then be modified in a second stage to include only the selected epigenetic and genetic biomarkers (including those from the scientific literature), representing less ROIs than in the default RRMS protocol. As less regions are targeted, more samples can be multiplexed, to maximize the sequencing resources of the flow cell, without a trade for coverage and accuracy. This two-step strategy with multiplexing facilitates biomarker discovery and further use in HBM studies with a high number of samples, at reduced cost. ^†^, chemical exposure assessed through the measurement of internal exposure biomarkers. ^¥^, the picture shows a GridION, an ONT sequencing instrument which can perform up to 5 sequencing runs in parallel with MinION flowcells. RNA fragments (and hence ncRNA epigenetic biomarkers) can also be measured with this device, although not simultaneously with DNAm and SNPs.

A promising method for DNAm analysis is the Reduced-Representation Methylation Sequencing (RRMS) from ONT. This approach allows the direct sequencing of raw DNA, as well as methylation detection, without harsh chemical pre-treatment and PCR amplification, making also the analysis less complex and time consuming. ONT is theoretically able to detect any DNA adducts, as long as a specific basecalling algorithm has been trained to detect the related modification, which is already the case for (hydroxy)methylated cytosine, methylated adenine and other proofs of concept (54, 55). Moreover, to produce comparable performance as the Infinium sequencing technology, ONT adaptive sampling (AS) is used to selectively sequence regions (customized in a database) containing CpG islands, while the other sequences are rejected by the nanopores and not sequenced (Figure 1) (56). As such, RRMS using AS with one single MinION flowcell (theoretical output capacity 50 Gb) aims to cover 100% of the human CpG islands with high sequencing depth (>15-20x), allowing accurate data interpretation with high confidence (57, 58). A recent study (59) concluded that ONT yielded better sensitivity, specificity and consistency, when compared to the gold standard Infinium MethylationEPIC array. Moreover, another comparison study showed that ONT long-read sequencing improved both the spatial resolution of DNAm and the discovery of new markers, because not limited by a predefined subset of probes from an array sequenced in short-reads (60). This improved spatial resolution can be particularly helpful for the measurement of DNAm in repetitive elements not covered by the Infinium arrays (61). AS is easily scalable and the database used for selective sequencing can be adapted to allow for the enrichment of any CpG regions. Moreover, SNPs can also be included with CpGs in the same database for AS, for a simultaneous analysis of genetic and DNAm biomarkers in one experiment, avoiding the use of one of the additional genotyping methods mentioned earlier (Figure 1). This combined analysis was already successfully employed in forensics and aging research (58, 62), as well as in cancer studies (63, 64). To the best of our knowledge, this was never applied to HBM, because this rapidly evolving technology was not mature enough for this application. However, considering its numerous advantages, ONT emerges now as a promising method for epigenetic studies (22), that should be included in HBM, as it fulfills the needs of measuring both SNPs and DNA modification (such as DNAm) biomarkers.

Single molecule, real time (SMRT) sequencing with Pacific Biosciences (PacBio) technology also offers the sequencing of long DNA fragment with detection of a limited number of base modification (such as methylation) with its kinetic signal analysis. However, PacBio sequencing offers a less flexible and scalable solution than ONT, with more complex sample preparation, resulting ultimately in a higher cost per sample (65, 66). Additionally, ONT encompasses more portable sequencing devices which can be interesting for HBM studies. Finally, real-time target selection with AS in ONT allows the enrichment for regions of interest, something that is not possible with PacBio.

ONT has still some limitations such as for instance read quality scores that have historically been lower compared to Illumina sequencing. Read quality is dependent on accurate signal measurement and basecalling algorithm performance, which can be affected by homopolymeric regions (67) or some methylation sites (68). However, continuous improvement is brought to the flowcell technology and chemistry, as well as to the basecalling algorithm. The last up to date R10 flowcells, V14 chemistry and dorado basecaller have been shown to produce accuracy (*q*-score > 20 and error rate < 1%) comparable to Illumina (67–70). Furthermore, with AS increasing sequencing depth, this guarantees that every nucleotide position and modification is called with high confidence for accurate data interpretation. Another limitation of the RRMS is the DNA input requirement (2 μg) for library preparation that is needed to obtain sufficient data output. Therefore, it requires sufficiently concentrated samples but this can be further optimized case by case with every matrix.

Finally, it should be noticed that other important epigenetic factors not covered in the present mini-review, such as small and long ncRNAs and their modifications, can also be measured with ONT (63). Alterations of ncRNAs due to environmental stressors, are gaining more and more interest as these molecules are known to mediate DNAm in physiological and pathological mechanisms (20). However, the direct sequencing of ncRNAs by ONT requires more complex and specific protocols (71). In particular, the sequencing of some small ncRNAs brings new challenges due to their very short length (15–50 bp) and the absence of polyA tail, which still requires further development to have a fully mature technology. Currently, it is not feasible to simultaneously sequence DNAm, SNP and ncRNA biomarkers through a single library preparation.

## 4 Challenges for a better integration of genetic and epigenetic biomarkers in large population studies

The biomarkers and related analytical methods are key aspects to carefully select during the design of large-population studies, because of cost and practical organization constraints, that can limit the number of participants. In the previous sections, we proposed DNAm and SNPs as promising effect and susceptibility biomarkers, respectively, that can be simultaneously measured by ONT. For large-population studies, ONT can still be costly, considering that one sample per MinION flowcell is preferred in EWAS to achieve adequate sequencing depth (>15-20x) and accuracy for data interpretation (60). Nevertheless, a cost effective two-step ONT strategy could be implemented, starting with a more costly open approach for marker discovery, and followed by a more affordable targeted method for marker validation (Figure 1). Few studies from Table 1 have adopted this approach, by using first Infinium arrays with a limited amount of samples for EWAS, and secondly pyrosequencing targeting few CpG sites in replication studies with more participants. Similarly, RRMS can be used in first intention with AS targeting all the CpG islands for marker discovery (open untargeted approach), and then be adjusted to detect only a set of selected biomarkers (targeted approach) during HBM studies (Figure 1). As less sequences are targeted for selective enrichment during this second stage, this can free sequencing resources in the MinION flowcell to allow multiplexing of several samples in one analysis, without a trade for coverage and accuracy. By sequencing only selected CpGs and SNPs, one MinION flow cell could handle multiple multiplexed samples at once, dramatically improving throughput and cost-efficiency. This multiplexing level can even be increased with the use of PromethION flowcells (theoretical capacity 290 Gb), encompassing 5 times more pores than MinION, and hence 5 times more sequencing capacity at similar cost.

Another critical aspect is the choice of the matrix where to measure the different biomarkers, i.e., exposure, effect and susceptibility. Among the studies listed in Table 1, urine was preferably used for the measurement of exposure biomarkers (except for PFAS), most of the time in combination with blood for the measure of effect and susceptibility biomarkers; although, sperm, placenta tissue and cord blood were also used depending on the biological question investigated. The sampling of blood is invasive and requires medical staff (unless remote blood sampling devices could be used (72)), which can limit the number of samples due to higher cost and participant reluctance. Alternatively, non-invasive samples, such as saliva, gained in popularity for HBM to measure effect biomarkers (7). Saliva is produced in abundance and is easy to collect. Furthermore, as it is mainly derived from intercellular fluid and blood, it contains key cellular and molecular components, such as a.o. leukocytes, buccal cells, microbial cells, inflammatory molecules, antibodies and hormones. Thanks to this, saliva is an interesting surrogate for effect and susceptibility biomarkers investigation, including DNAm and SNPs, as high quality DNA can be extracted from the cells present in saliva (73). Saliva can be combined with urine collection (for exposure biomarkers) for a full non-invasive sampling. Nonetheless, caution must be taken when measuring DNAm in different fluids, as some methylation marks are tissue and cell dependent (74, 75). This heterogeneity is already accounted for in DNAm studies using blood, where data correction is applied according to cell composition, estimated using deconvolution methods (76, 77) and reference panels (78) (Table 1). Saliva is a slightly more challenging matrix, as it also contains (dead) buccal cells as well as microorganisms, and has more inter-individual variability. However, this should not be limiting, as reference-based deconvolution methods can be used (74, 79) with saliva reference panels to account for cellular heterogeneity (80). Concerning non-human DNA, it can be aimed to be ignored by using AS included in the RRMS protocol, as only human CpGs are enriched. Some studies compared DNAm profiling in saliva vs. blood, and concluded that despite some differences, there was an overall good correlation between the two matrices, with key common biomarkers present in both sample types (5, 81–83). Considering this, it is encouraged to do marker discovery with EWAS directly in saliva, and if needed to test for biomarker replication in blood with targeted methods, to ensure that the same changes can be detected in both fluids, before assuming interchangeability. The evidence reviewed here provides proofs of concept that saliva (or eventually other non-invasive human samples) can deliver high quality DNA, suitable for DNAm and SNP biomarkers measurement. Nevertheless, more studies are needed to explore the use of saliva in identifying and measuring these biomarkers in the context of environmental exposure and HBM, as proposed in Figure 1.

## 5 Conclusion

Amongst the different epigenetics mechanisms, DNA-methylation is a promising effect biomarker that should be combined with susceptibility genetic biomarkers and exposure biomarkers, to provide the full and accurate picture in large population HBM studies, for improved risk assessment. However, the collection and analysis of thousands of samples in exposure studies are still costly and practically challenging. In this study, we presented how ONT could be a method of choice for simultaneous analysis of genetic (SNP) and epigenetic (DNAm) markers, with potential to include a large panel of DNA modifications in the future. While ONT has mainly be used in medical research, the most recent improvements in accuracy (Q20+) and selective sequencing (AS) make this technology mature to be used in HBM, addressing the limitations of large-population (epi)genetic studies. Indeed, this scalable method can first be used with a small subset of samples in EWAS for marker discovery, and later in a large population study with sample multiplexing at reduced cost and increased throughput for HBM. Moreover, saliva appears to be a valid alternative to blood for biomarker measurement, facilitating sample collection from a high number of participants. The use of ONT and non-invasive samples, for combined measurement of effect and susceptibility biomarkers, will facilitate the evaluation of the impact of environmental exposure, and will contribute to a better understanding of the AOPs. This will eventually lead to a NGRA for chemicals as aimed for in the PARC project to protect human health and the environment, with amongst others new innovative approaches (8).

## Footnotes

^1^ https://www.eu-parc.eu
